# Residual Adhesive Removal Methods for Rebonding of Debonded Orthodontic Metal Brackets: Systematic Review and Meta-Analysis

**DOI:** 10.3390/ma14206120

**Published:** 2021-10-15

**Authors:** Guillermo Grazioli, Louis Hardan, Rim Bourgi, Leina Nakanishi, Elie Amm, Maciej Zarow, Natalia Jakubowicz, Patrycja Proc, Carlos Enrique Cuevas-Suárez, Monika Lukomska-Szymanska

**Affiliations:** 1 Department of Dental Materials, School of Dentistry, Universidad de la República. Av. General Las Heras 1925, Montevideo 11300, Uruguay; ggrazioli@odon.edu.uy; 2 Department of Restorative Dentistry, School of Dentistry, Saint-Joseph University, Beirut 1107 2180, Lebanon; louis.hardan@usj.edu.lb (L.H.); rim.bourgi@net.usj.edu.lb (R.B.); 3 Graduate Program in Dentistry, School of Dentistry, Federal University of Pelotas, Rua Gonçalves Chaves, 457, Pelotas 96015560, Brazil; leinaa_@hotmail.com; 4 Department of Orthodontics, School of Dental Medicine, Saint Joseph University, Beirut 1107 2180, Lebanon; elie.el-amm@usj.edu.lb; 5 “NZOZ SPS Dentist” Dental Clinic and Postgraduate Course Centre—pl. Inwalidow 7/5, 30-033 Cracow, Poland; dentist@dentist.com.pl (M.Z.); nljakubowicz@gmail.com (N.J.); 6 Department of Pediatric Dentistry, Medical University of Lodz, Pomorska 251, 92-213 Lodz, Poland; patrycja.proc@umed.lodz.pl; 7 Dental Materials Laboratory, Academic Area of Dentistry, Autonomous University of Hidalgo State, Circuito Ex Hacienda La Concepción S/N, San Agustín Tlaxiaca 42160, Mexico; 8 Department of General Dentistry, Medical University of Lodz, 251 Pomorska St., 92-213 Lodz, Poland

**Keywords:** adhesive, bonding, bracket

## Abstract

Debonding of orthodontic brackets is a common occurrence during orthodontic treatment. Therefore, the best option for treating debonded brackets should be indicated. This study aimed to evaluate the bond strength of rebonded brackets after different residual adhesive removal methods. This systematic review and meta-analysis was conducted according to the Preferred Reporting Items for Systematic Reviews and Meta-Analyses (PRISMA) statement. PubMed, Web of Science, The Cochrane Library, SciELO, Scopus, LILACS, IBECS, and BVS databases were screened up to December 2020. Bond strength comparisons were made considering the method used for removing the residual adhesive on the bracket base. A total of 12 studies were included for the meta-analysis. Four different adhesive removal methods were identified: sandblasting, laser, mechanical grinding, and direct flame. When compared with new orthodontic metallic brackets, bond strength of debonded brackets after air abrasion (*p* = 0.006), mechanical grinding (*p* = 0.007), and direct flame (*p* < 0.001) was significantly lower. The use of an erbium-doped yttrium aluminum garnet (Er:YAG) laser showed similar shear bond strength (SBS) values when compared with those of new orthodontic brackets (*p* = 0.71). The Er:YAG laser could be considered an optimal method for promoting the bond of debonded orthodontic brackets. Direct flame, mechanical grinding, or sandblasting are also suitable, obtaining clinically acceptable bond strength values.

## 1. Introduction

The effectiveness of fixed orthodontic treatment requires an adequate bonding between brackets and enamel surfaces [1]. Orthodontic brackets are fixed appliances that are bonded to the tooth and should remain in place until the end of treatment [2], to achieve this, the bond strength between bracket base and enamel surfaces should be strong enough to resist orthodontic forces and masticatory loads [3]. In this sense, many factors can lead to bracket–enamel bond failure, including the type of enamel conditioner, composition of adhesive, bracket base design, bracket material, as well as clinician skills [4].

Debonding of orthodontics from teeth is a common occurrence during orthodontic treatment, varying between 1.8% [5] and 20.1% [6]. Debonding of brackets during treatment is an unpleasant occurrence for the clinician and the patient resulting in increased treatment costs and duration [7]. During orthodontic treatment, the clinician may decide to debond one bracket intentionally and to rebond it on the tooth in a better position [8]. Therefore, clinicians have often to deal with what is the best option for treating with unintentional/intentional debonded brackets, and regardless of the cause of debonding, the orthodontist must decide whether to rebond the same bracket or to bond a new one [9].

One solution is to recycle or re-condition these brackets to reuse them for the same patient during the same visit. The re-condition process consists of removing bonding agent remnants from the bracket base, thus allowing the brackets to be rebonded [10]. Once a bracket is rebonded for its use again, it should exhibit sufficient bond strength. Thus, the main challenge in rebonding brackets is restoring the bracket base to a retentive pattern without damaging the bracket itself [11].

Adhesive remnants of the dislodged brackets had been conventionally removed in-office by using green stones [12], direct flame [13], tungsten-carbide bur [14], sandblasting [15], silica coating [16], or laser application [17]. Even though these methods can be easily performed out in the dental office with minimal cost, there is no consensus as to which is the best method to remove adhesive remnants from the bracket base. Accordingly, this systematic review and meta-analysis aims to evaluate the bond strength of rebonded brackets after different residual adhesive removal methods. The hypothesis to be tested is that different residual adhesive removal methods would provide similar bond strength of recycled/reused brackets when compared to new orthodontic brackets.

## 2. Materials and Methods

This systematic review and meta-analysis was reported by following the guidelines of the PRISMA statement [18]. The following PICOS framework was used: population, debonded orthodontic brackets; intervention, methods for residual adhesive removal; control, new orthodontic brackets; outcomes, bond strength; and study design, in vitro studies. The research question was: is there an optimal method to remove the residual adhesive of debonded orthodontic brackets?

### 2.1. Literature Search

The literature search was performed by two independent reviewers until 15 December 2020. The following five electronic databases were screened: PubMed (MedLine), ISI Web of Science, Cochrane Library, SciELO, and Scopus. The search strategy used is listed in Table 1. The reviewers also hand-searched the reference lists of included articles for the identification of additional manuscripts. After the initial screening, all studies were imported into Mendeley Desktop 1.17.11 software to remove duplicates.

### 2.2. Study Selection

Two reviewers independently assessed the titles and abstracts of all the manuscripts. Manuscripts for full-text review were selected according to the following eligibility criteria: (1) evaluated the bond strength of new orthodontic metallic brackets; (2) evaluated the bond strength of debonded orthodontic metallic brackets after using a method to remove the adhesive of the orthodontic metallic bracket base; (3) evaluated the bond strength of debonded orthodontic metallic bracket on new intact enamel; (4) included mean and standard deviation data in MPa; (5) published in the English language. Case reports, case series, pilot studies, and reviews were excluded. Full copies of all the potentially relevant studies were analyzed. Those that appeared to meet the inclusion criteria or had insufficient data in the title and abstract to make a clear decision were selected for full analysis. The full-text papers were independently assessed by two authors. Any disagreement regarding the eligibility of the included studies was resolved through discussion and consensus by a third reviewer.

### 2.3. Data Extraction

Data of interest from the manuscripts included was extracted using Microsoft Office Excel 2019 sheets (Microsoft Corporation, Redmond, WA, USA). These data included the year of publication, country, type of bracket, type of tooth, orthodontic adhesive used, the method for adhesive removal, the mean and standard deviation of the bond strength, and storage conditions.

### 2.4. Quality Assessment

The methodological quality of each study was assessed by two reviewers, according to the parameters of a previous systematic review of in vitro studies [19]. The risk of bias in each article was evaluated according to the description of the following parameters: specimen randomization, single-operator protocol implementation, blinding of the operator, the presence of a control group, standardization of the sample preparation, adhesive remnant index evaluation (ARI), use of all materials according to the manufacturer’s instructions, and description of the sample size calculation. If the authors reported the parameter, the study received a “YES” for that specific parameter. In case of missing information, the parameter received a “NO.” The risk of bias was classified according to the sum of “YES” answers received: 1 to 3 indicated a high bias, 4 to 6 medium, and 7 to 8 indicated a low risk of bias.

### 2.5. Statistical Analysis

Meta-analyses were carried out by using a software program (Review Manager Software version 5.4, The Cochrane Collaboration, Copenhagen, Denmark). The analyses were carried out using a random-effect model, and pooled-effect estimates were obtained by comparing the mean difference between bond strength values obtained using new orthodontic brackets or after removing the adhesive resin. Bond strength comparisons were made considering the method used for removing the residual adhesive on the bracket base. A *p*-value < 0.05 was considered statistically significant. Statistical heterogeneity of the treatment effect among studies was assessed using the Cochran Q test and the inconsistency I^2^ test.

## 3. Results

**Figure 1:**
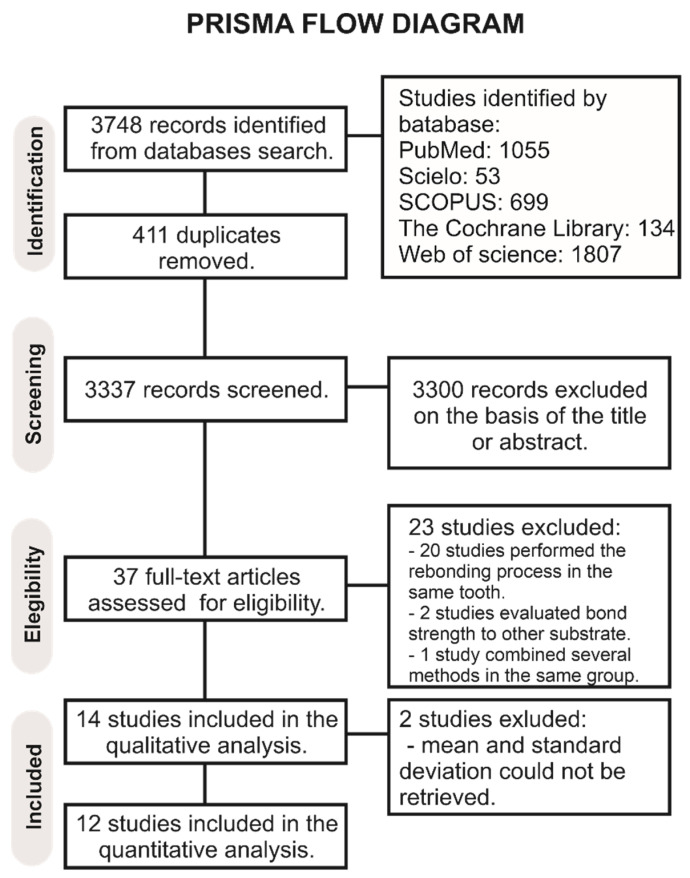
Prisma flow diagram of the study.

A total of 3748 publications were collected from all databases (Figure 1).

After duplicates were removed, the literature review yielded 3337 manuscripts for initial examination. From these studies, 3300 studies were excluded after reviewing their titles and summaries. In total, 37 studies were examined by full-text reading. Of these studies, 23 were not included in the qualitative analysis: 2 studies evaluated the bond strength to other substrates different than enamel [20,21], 1 study combined several methods for adhesive removal in the same group [22], and 20 studies performed the rebonding process in the same tooth where the initial bonding process was performed [9,10,12,23,24,25,26,27,28,29,30,31,32,33,34,35,36,37,38,39], of the remaining 14 studies, 2 were excluded from the quantitative analysis because the mean and standard deviation was not available [40,41], totalizing 12 studies for the quantitative analysis.

Four different adhesive removal methods were identified in this review. These included air abrasion [13,14,15,17,42,43,44,45,46,47], laser [17,42], mechanical grinding [14,43,44], and direct flame [12,41,43]. The characteristics of these studies are summarized in Table 2.

**Figure 2:**
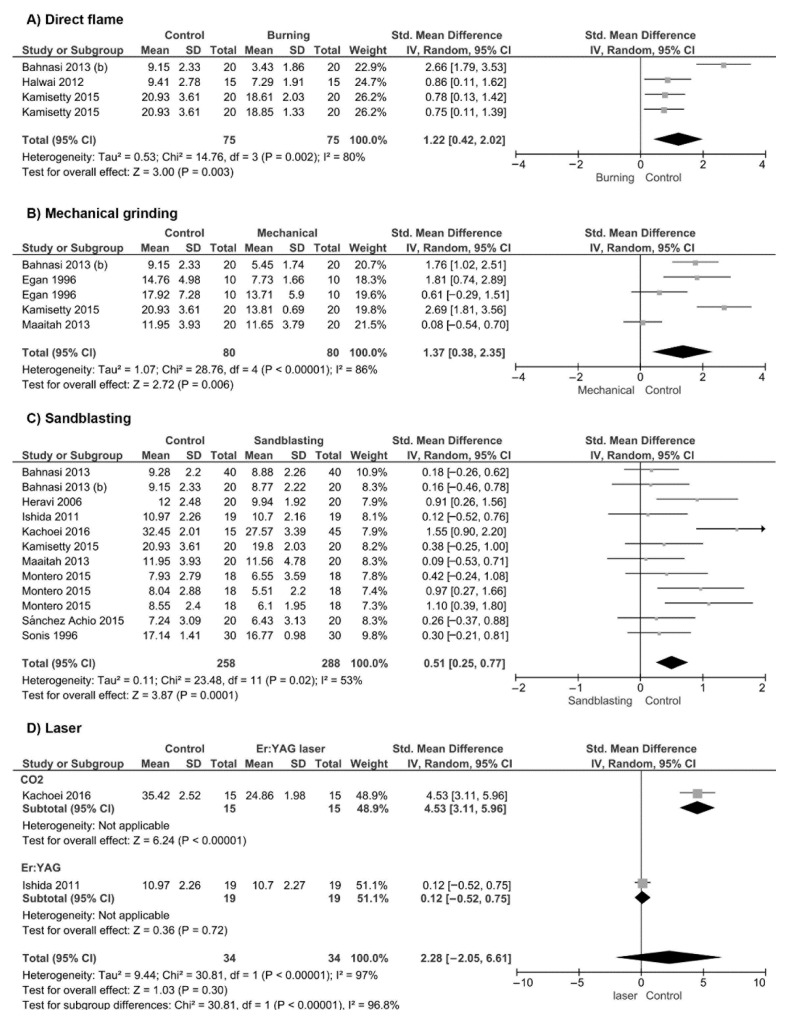
Results of the meta-analysis of bond strength of debonded orthodontics brackets after residual adhesive removal using; (**A**) Direct flame; (**B**) Mechanical grinding; (**C**) Sandblasting; and (**D**) Laser.

A meta-analysis was performed with 12 in vitro studies. Separate analyses for each adhesive removal method were performed (Figure 2). As the control for each study, the SBS value of new orthodontic brackets was considered. Direct flame methods for removing the residual adhesive were evaluated (Figure 2A). The meta-analysis demonstrated that these methods achieved significantly lower bond strength values of rebonded brackets when compared with the new bonded brackets (*p* < 0.001). With regards to the use of mechanical grinding methods to remove the residual adhesive from the base of orthodontic brackets, significantly lower SBS values were also observed (Figure 2B; *p* = 0.007). SBS of rebonded orthodontic brackets after adhesive removal with sandblasting was analyzed (Figure 2C). The meta-analysis performed demonstrated that bond strength values after adhesive removal through sandblasting were significantly lower than the bond strength of new orthodontic brackets (*p* = 0.006). With regards to the use of laser, two different types of laser were identified (Figure 2D). When a CO_2_ laser was used for adhesive removal, the SBS of rebonded brackets was lower than the bond strength of new orthodontic brackets (*p* < 0.001). On the other hand, the use of an Er:YAG laser for adhesive removal showed similar SBS values when compared with those of new orthodontic brackets (*p* = 0.71).

According to the parameters considered in the risk of bias assessment, the majority of studies were classified with a medium risk of bias (Table 3). Several of the studies failed to report single-operator, operator-blinded, and sample size calculation parameters.

## 4. Discussion

This systematic review and meta-analysis aimed to evaluate the bond strength of debonded brackets after different residual adhesive removal methods. Direct flame, mechanical grinding, sandblasting, and laser were the methods found in the literature used for this purpose. Except for the Er:YAG laser, none of the methods evaluated managed to restore SBS values of new orthodontic brackets values, thus our hypothesis was partially rejected.

One of the methods proposed to remove the adhesive remnant after bracket debonding is direct flaming of the bracket base. Under the use of this method, removal of the bonding agent is the most critical part of the recycling process and requires long exposure to heat [44]. The results of the present meta-analysis helped to demonstrate that this method was unable to recover the original values achieved by new orthodontic brackets. Several explanations may be suggested to explain this behavior. First, direct flaming increases the temperature of the bracket base to a temperature in the range of 600–800 °C, which can lead to the disintegration of the metal alloy, and consequently weakens its structure, making it more vulnerable to damage [30]. Also, as most of the metallic orthodontic brackets are made of austenitic stainless steel, application of heat leads to the formation of chrome-carbide compounds, which can render them more susceptible to tarnish and corrosion, and this, in turn, could be responsible for its failure in the mouth [48]. Finally, it has been found that the heat treatment could lead to a decrease in the diameter of the support mesh, which is caused by the presence of large amounts of adhesive residues on the base [30].

When observing the data about mechanical grinding, four studies reported this method. For this purpose, a green stone [43,48], or a carbide bur [14,41] at slow speed were used to grind the bracket surface. The meta-analysis revealed that, when this method was used for the removing of adhesive residual, significantly lower values in the bond strength of rebonded brackets were achieved. During the adhesive removal from the bracket, the preservation of the integrity of the bracket mesh is crucial to ensure an adequate bond strength to the enamel. By grinding the bracket base using a green stone or a carbide bur, there is a high risk of damage or grinding off the mesh base itself, resulting in a decrease in bond strength. [12] Also, grinding the bracket mesh has been proved to leave a considerable amount of the adhesive, obliterating the mesh and decreasing the contact area, thus eliminating virtually any mechanical retention [10,12,14,44].

Sandblasting has been described as a viable procedure for rebonding accidentally lost brackets. This method was the most used in the studies included in the present systematic review. The findings obtained by the meta-analysis suggest that the bond strength observed by debonded, cleaned brackets with sandblasting is significantly lower when compared with new brackets. Previous research has demonstrated that sandblasting of the bracket base could provoke distortion of the mesh [33]. In this sense, the air abrasion procedure causes macro and microscopic alterations in the structure of the bonding surface, consequently affecting the bond strength outcomes [12]. Also, it has been described that after sandblasting, some abrasive particles adhere to the blasted surface, and it is possible that bond strength between any luting material and the abrasive particle remnants might exceed the bond strength of the abrasive particles and the bonding surface, causing premature debonding [49]. On the other hand, the sandblasting process is not able to remove all the resin attached to the bonding mesh [17], directly affecting the bond strength outcomes.

When observing the data about lasers, two different methods were analyzed separately [17,43]. This technology selectively ablates composite by high pulse repetition rates [49]. When analyzing the CO_2_ laser, it was found that it is not a suitable method for recycling brackets because considerable amounts of adhesive remnants were left on the base of CO_2_ laser-irradiated brackets [49]. As explained before, the remaining adhesive on the bracket base lessens the contact area between the meshwork and adhesive and leads to a decrease in bond strength values. On the other hand, the analysis of the results from the Er:YAG laser method demonstrated that this method is efficient for removing the residual adhesive, being that the values obtained were similar to those of new orthodontic brackets. This result could be explained due to the complete removal of the residual adhesive from the bracket bases without altering the micro and macrostructure of the mesh, resembling the appearance and bonding performance of new brackets [17]. Nevertheless, it should be advised that the use of the Er:YAG laser could melt the meshwork of the bracket base due to heat, and some precautions should be taken when using this method [17].

Regarding the limitations of this systematic review, it is important to highlight that all analyses performed showed high heterogeneity values, which could be attributed to the lack of standardization of the methods for evaluation of the SBS; actually, none of the included studies indicated the following of the international standards for bond strength tests to dental tissues. Future research with more standardized methods is desired to reduce the heterogeneity between the studies focused on this topic and also to establish the optimal protocol for the adhesive removal for rebonding of debonded orthodontic brackets. Also, it is important to encourage researchers for designing and conducting clinical trials evaluating this outcome.

On the other hand, it should be pointed out that despite the meta-analysis showing statistical differences between the SBS of debonded and new brackets, such differences are not clinically relevant, this is because the mean bond strength values of the methods evaluated succeeded to achieve at least 6 MPa, which is the minimum bond strength required for successful orthodontic treatment [17,23]. This could also lead to the perspective that rebonding of debonded orthodontic brackets in the same patient is a reliable treatment option, as long as the adhesive residual within the orthodontic base is completely removed using the above-mentioned procedures.

## 5. Conclusions

Within the limitations of this systematic review, it could be concluded that the Er:YAG laser could be considered as an optimal method for promoting the bond of debonded orthodontic brackets, this conclusion is based on the fact that the bond strength of rebonded orthodontic brackets was the same as that of the new brackets. Nevertheless, the data suggest that the use of direct flame, mechanical grinding, or sandblasting are suitable options for the removal of residual adhesive from the orthodontic bracket base, obtaining clinically acceptable bond strength values.

## Figures and Tables

**Table 1:** Keywords used in the search strategy.

Search Strategy	
# 1	Orthodontic bracket OR bracket OR braces OR stainless steel bracket OR recycled bracket.
# 2	Rebonded OR rebonding OR reconditioning OR recycling OR recycling methods OR recycled brackets OR rebonded brackets OR electropolishing OR sandblasting OR ultrasonic scaling OR heating OR Er:YAG laser OR CO2 laser
# 3	#1 and #2

**Table 2:** Demographic data of included studies.

Study	Bracket Used	Tooth Used	Orthodontic Adhesive Used	Storing Conditions	Residual Adhesive Removal Method Used	Secondary Outcome
Achio, 2015	Stainless-steel premolar bracket (Unitek^TM^ Gemini Bracket, 3M Unitek, Monorovia, CA, USA)	Human premolar	Transbond™ Plus Self Etching Primer (3M Unitek)/Transbond^TM^ XT Light Cure Composite (3M Unitek)	Thermocycling (500 cycles between 5 °C and 55 °C)	Sandblasting (Al_2_O_3_; 50 µm, 90 psi, 10 mm, 10–15 s)	Adhesive remnant index
Bahnasi, 2013	Stainless steel upper premolar bracket (Unitek^TM^ Gemini Bracket (3M Unitek)	Human premolar	Light Cure Orthodontic Adhesive Primer (3M Unitek)/Transbond^TM^ XT Light Cure Composite (3M Unitek)	Thermocycling (500 cycles between 5 °C and 55 °C)	Sandblasting (Al_2_O_3_; 50 µm, 90 psi, 10 mm, 20–30 s)	Adhesive remnant index
Bahnasi, 2013 (b)	Stainless steel upper premolar bracket (Unitek^TM^ Gemini Bracket, 3M Unitek)	Human premolar	Light Cure Orthodontic Adhesive Primer (3M Unitek)/Transbond^TM^ XT Light Cure Composite (3M Unitek)	Thermocycling (500 cycles between 5 °C and 55 °C)	Sandblasting (Al_2_O_3_; 50 µm, 90 psi, 10 mm, 20–30 s). Mechanical grinding with a carbide bur with high-speed hand piece. Direct flame with a gas torch flame for 5 s.	Qualitative analysis of the distortion of the base with SEM
Egan, 1996	Stainless steel upper premolar brackets (GAC International Inc., New York, NY, USA)	Human premolar	Rely a Bond (Reliance Orthodontic Products Inc., Itasca, IL, USA) and Phase II paste-paste (Reliance Orthodontic Products Inc.)	Distilled water at 37 °C for 1 week	Mechanical grinding with a green stone	Failure mode
Harini, 2011	Stainless steel premolar brackets *	Human premolar	All Bon-2 (Bisco Inc., Schaumburg, IL, USA.	Distilled water for 24 h	Direct flame with a soldered torch for 5 s.	Adhesive remnant index
Heravi, 2006	Standard Edgewise metal brackets (Dentarum Corp., Ispringen, Germany)	Human upper premolar	No-mix composite (Dentarum Corp., Germany)	Distilled water at 37 °C for 48 h	Mechanical grinding with a tungsten carbide bur with high-speed hand piece	Adhesive remnant index
Ishida, 2011	Metal premolar bracket (Unitek^TM^ Victory series, 3M Unitek)	Human premolar	Transbond™ Plus Self Etching Primer (3M Unitek)/Transbond^TM^ XT Light Cure Composite (3M Unitek)	Artificial saliva at 37 °C for 24 h	Er,Cr:YSGG laser (Power output of 3.75 W, wavelength of 2.78 µm, a pulse duration of 140 µs, a frequency of 20 Hz, and air and water levels, each 50%)	Adhesive remnant index
Kachoei, 2016	Maxillary central incisors (Ortho-Organizer, Carlsbad, CA, USA)	Bovine upper central incisors	Unite Bonding System (3M Unitek, USA)	Distilled water at 37 °C for 1 week	Sandblasting (Al_2_O_3_; 50 um, 5 mm). CO_2_ laser (wavelength of 10,600 nm and a 3 W output power, for 15 s)	Adhesive remnant index
Kamissety, 2015	Stainless steel lower premolar brackets (Gemini, 3M Unitek)	Lower human premolar	Transbond XT adhesive (3M Unitek).	Artificial saliva for 24 h at 37 °C	Mechanical grinding with a green stone with low-speed hand piece. Sandblasting (Al_2_O_3_, 50 µm, 10 mm, 90 PSI) Direct flaming with a micro torch Direct flaming with a Bunsen flame	UV/Vis transmittance analysis
Maaitah, 2013	Premolar brackets (Omni 0.022′’ Roth, GAC International Inc, New York, NY, USA)	Human premolar teeth	Transbond^TM^ XT Adhesive (3M Unitek)	Thermocycling (500 cycles between 5 °C and 55 °C)	Mechanical grinding with slow speed round tungsten carbide bur. Sandblasting (CoJetTM System Set; 3M Espe)	Adhesive remnant index
Montero, 2015	Upper central incisor brackets (Unitek^TM^ Victory series, 3M Unitek)	Bovine upper central incisors	Transbond Plus Self Etching Primer (3M Unitek)/Transbond XT (3M Unitek)	Distilled water at 37 °C for 24 h	Sandblasting (Al_2_O_3_; 25 µm, 50 µm, or 110 µm at 5 mm)	SEM observation
Shahamfar, 2014	Premolar bracket (Equilibrium, Dentaaurum Inc., Ispringen, Germany)	Human premolar teeth	Light Bond^TM^ (Reliance Orthodontic products, IL, USA)	Distilled water at 37 °C for 24 h	Mechanical grinding with slow speed multi blade tungsten carbide bur.	Adhesive remnant index
Sonis, 1996	Lower premolar brackets (GAC International, Inc., Central Islip, Long Island, NY, USA)	Lower human premolar	Rely-a-bond (Reliance, Inc., Itasca, IL, USA)	Thermocycling (1000 cycles between 10 °C and 50 °C)	Sandblasting (90 µm; 90 PSI, 15 to 30 s)	Scanning electron micrograph of base surface
Wheeler, 1983	Stainless steel premolar brackets	Human premolar	Dyna Bond II Series B (Unitek Corporation, Monrovia, CA, USA)	Non-specified	Heating in an oven for 50 min at 454 °C	

**Table 3:** Qualitative synthesis (risk of bias assessment).

Study	Specimen Randomization	Single Operator	Operator Blinded	Control Group	Standardized Specimens	ARI	Manufacturer’s Instructions	Sample Size Calculation	Risk of Bias
Achio, 2015	Yes	No	No	Yes	Yes	Yes	Yes	No	Medium
Bahnasi, 2013	No	No	No	Yes	Yes	Yes	Yes	No	Medium
Bahnasi (b), 2013	Yes	No	No	Yes	Yes	No	Yes	No	Medium
Egan, 1996	No	No	No	Yes	Yes	Yes	Yes	No	Medium
Harini, 2011	No	No	No	Yes	Yes	Yes	Yes	No	Medium
Heravi, 2006	No	No	No	Yes	Yes	Yes	Yes	No	Medium
Ishida, 2011	Yes	Yes	No	Yes	Yes	Yes	Yes	No	Medium
Kachoei, 2016	Yes	Yes	Yes	Yes	Yes	Yes	Yes	Yes	Low
Kamissety, 2015	No	No	No	Yes	Yes	No	Yes	No	High
Maaitah, 2013	Yes	Yes	No	Yes	Yes	Yes	Yes	No	Medium
Montero, 2015	Yes	No	No	Yes	Yes	No	Yes	Yes	Medium
Shahamfar, 2014	No	No	No	Yes	Yes	Yes	Yes	No	Medium
Sonis, 1996	Yes	No	No	Yes	Yes	No	Yes	No	Medium
Wheeler, 1983	Yes	Yes	No	Yes	Yes	No	Yes	No	Medium

## Data Availability

The data that support the findings of this study are available from the corresponding author upon reasonable request.
